# Increasing utilization of urethroplasty for male urethral stricture disease: analysis of in-hospital interventions in Germany from 2006 to 2023

**DOI:** 10.1007/s11255-025-04487-1

**Published:** 2025-04-28

**Authors:** Roman Herout, Luka Flegar, Juliane Putz, Nicole Eisenmenger, Johannes Huber, Christian Thomas, Martin Baunacke

**Affiliations:** 1 Department of Urology, Faculty of Medicine and University Hospital Carl Gustav Carus, TUD Dresden University of Technology, Fetscherstraße 74, 01307 Dresden, Germany; 2 Department of Urology, Philipps-University Marburg, Marburg, Germany; 3 Department of Urology, Royal Melbourne Hospital, Melbourne, Australia; 4 Reimbursement Institute, Hürth, Germany; 5 Department of Urology, University Hospital Heidelberg, Heidelberg, Germany

**Keywords:** Urethral stricture disease, Internal urethrotomy, Urethral dilatation, Buccal mucosa graft urethroplasty

## Abstract

**Purpose:**

There is a wide range of surgical treatments for urethral strictures. There is no information on the extent to which these procedures have been used over the past few years.

**Methods:**

We analyzed data from Diagnosis Related Groups (DRG) billing data from DESTATIS. Linear regression models were utilized for the analysis of trends over time.

**Results:**

A significant decrease of male urethral stricture cases during the study period was observed. Cases decreased from 48,020 cases in 2006 to 33,062 cases in 2023, i.e. -31%. This decline was mainly driven by the decrease in cases in the younger age group (≤ 79 years), whereas a significant increase was noted in octogenarians (+ 24%). A total of 834,476 procedures for urethral stricture disease were analyzed. In accordance with total case numbers, inpatient surgical interventions for urethral stricture disease decreased by 39% during the study period. Internal urethrotomy significantly decreased by 50% (from 41,443 in 2006 to 20,777 in 2023; p < 0.001). Likewise, urethral dilatations significantly decreased by 17% (from 14,134 in 2006 to 11,674 in 2023; p = 0.002). On the contrary, urethroplasties significantly increased by 156% (from 873 in 2006 to 2,239 in 2023; p < 0.001).

**Conclusion:**

We present contemporary data on urethral stricture disease management in Germany. Case numbers of internal urethrotomies and urethral dilatations are declining while urethroplasty is increasingly utilized as a definitive treatment for urethral stricture disease. This may reflect an optimized treatment of urethral strictures with more urethroplasties and thus fewer relapses to be treated.

**Supplementary Information:**

The online version contains supplementary material available at 10.1007/s11255-025-04487-1.

## Introduction

Urethral stricture disease (USD) is defined as a narrowing of the urethral lumen due to scarring that leads to a functional obstruction of the lower urinary tract, which in turn negatively affects overall quality of life [1]. Patients typically suffer from lower urinary tract symptoms (LUTS) and long-term sequelae such as irreversible damage to the urinary tract with impairment of renal function are dreaded complications [1]. USD is more common in male than in female and affects about 0.6–0.9% of the population [2, 3]. Urethritis, mainly caused by Neisseria gonorrhoe, was the main cause of USD up until the mid of the twentieth century when penicillin was discovered [4]. However, nowadays iatrogenic causes are responsible for about 45% of all strictures [5, 6]. In younger patients strictures are mainly due to idiopathy, hypospadias surgery or pelvic fracture [5, 6]. Generally, the incidence of USD increases with age [2]. Various interventions for USD have been developed which are aimed at widening the urethra as to restore unimpaired urine flow [4]. However, USD is a malady that tends to recur with worse outcomes the longer the stricture and the more interventions had been performed [1]. Direct visual internal urethrotomy (DVIU) and urethral dilatation are the most common procedures for USD. Unfortunately, recurrence rates after DVIU and urethral dilatation are very high and range between 40 and 92% [7–9]. Open surgery, performed as urethroplasty, on the other hand is an option for patients with high success rates (> 85%) [10–12]. Various urethroplasty techniques have been described with excision with primary anastomosis and urethroplasty with buccal mucosa graft being the most commonly performed procedures. In complex stricture disease a two or multiple-staged approach is sometimes necessary.

There are no published population-based data on urethral stricture disease management in Germany to our knowledge. In our personal experience, the ambition to perform urethroplasties as a definitive treatment whenever possible, is clinically often challenged by the aging population with substantial comorbidities. Since there are no specific reconstructive fellowships in Germany and basically no structured curricula to learn urethroplasty, it was crucial for us to assess whether urethroplasties for USD are declining in Germany. Therefore we aimed to assess current treatment patterns for USD in adults and to present population-based data from Germany on operative interventions for USD over an 18-year period.

## Materials and methods

### German hospital billing database (diagnosis related groups)

Since 2004, reimbursement of inpatient care in Germany has been based on an adapted version of the international diagnosis-related group coding of diagnoses according to ICD-10 and medical procedures according to a German version of the international classification of operations and procedures. Diagnoses and procedures of each hospital case are submitted annually to the Institute for Hospital Reimbursement and then to the Federal Statistical Office (DESTATIS). The data covers all hospitals that bill according to the DRG remuneration system. Excluded are hospitals in penal or forensic institutions and police hospitals, psychiatric facilities and institutes for psychosomatics and psychotherapeutic medicine. We performed a total population analysis from 2006 to 2023 with the analysis tool “reimbursement.INFO” (Reimbursement Institute, Hurth, Germany).

Hospitals and other inpatient providers are reimbursed via the diagnosis related groups (DRG). A specific DRG code is comprised of an ICD-10 (International Statistical Classification of Diseases and Related Health Problems) diagnosis code and an OPS (German adaption of the International Classification of Procedures in Medicine) code for the performed intervention. Specific ICD-10 codes for USD management were analyzed (Suppl. Tbl. 1 & 2). Age groups in this data set are divided into five-year groups, which is why the survey of adult men starts at the age of 20.

### Data analysis, protection and ethics statement

We followed the "REporting of studies Conducted using Observational Routinely collected health Data" (RECORD) statement and performed all actions in accordance with the Declaration of Helsinki in its latest version [13]. Due to the nature of the data (billing data from DESTATIS), ethical approval was not needed. Statistical analysis was performed using t-tests and chi-square tests, with a significance level set at p < 0.05. Linear regression models were utilized for the analysis of trends over time. All calculations were performed with “IBM SPSS Statistics 28” (Armonk, NY, USA).

## Results

### Diagnosis of urethral strictures in German hospitals

**Fig. 1:**
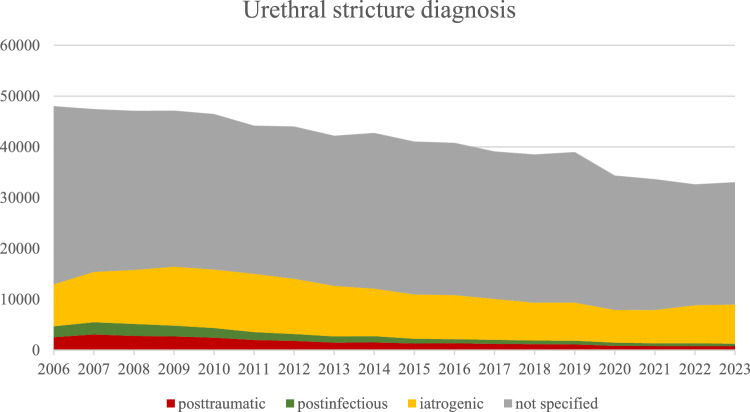
Urethral stricture diagnosis (main & secondary diagnosis) in German hospitals from 2006–2023

We observed a decline of the diagnosis “urethral stricture” (coded as N35.0. N35.1, N35.8, N35.9, N99.1) in German hospitals in adult men ≥ 20 years from 2006 to 2023 by 31% (from 48,020 to 33,062 coded cases; -959.4 ± 48.2 diagnosis/year; p < 0.001). This trend is depicted in Fig. 1. Regarding the distribution of the etiology of the USD as part of a primary diagnosis, the following development can be seen: significant decrease by 64% of posttraumatic strictures (from 1155 to 416 cases; − 55.4 ± 4.2 diagnosis/year; p < 0.001), significant decrease by 84% of postinfectious strictures (from 759 to 125 cases; − 42.7 ± 3.8 diagnosis/year; p < 0.001), non-significant decrease in iatrogenic strictures (from 3927 to 4133 cases, − 114.6 ± 30.7 diagnosis/year; p = 0.002) and a significant decrease by 33% of not specified strictures (from 10,228 to 6828 cases, − 106.7 ± 37.4 diagnosis/year; p = 0.01). These developments are visualized in the area chart of Fig. 1.

**Fig. 2:**
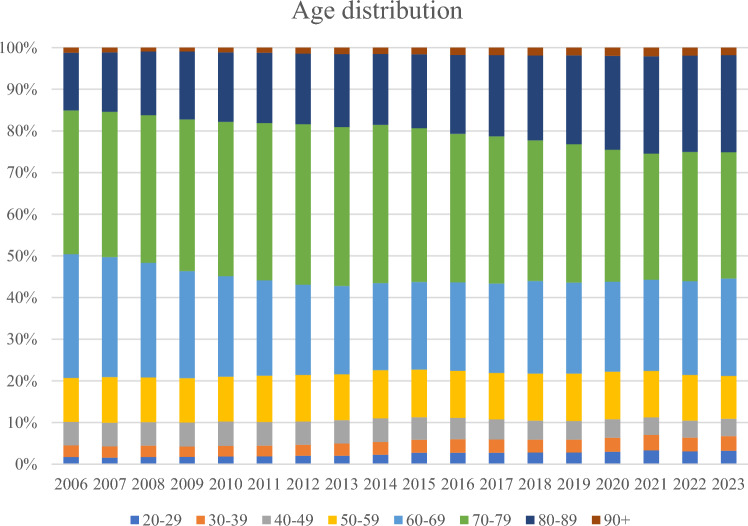
Development of age distribution in urethral stricture surgery in Germany from 2006–2023

The age distribution of USD (as the main diagnosis) is depicted in Fig. 2 and shows the following development: non-significant decrease in patients aged 20–39 years by 15% (from 1437 to 1225 cases; − 4.3 ± 4.9 diagnosis/year; p = 0.4), significant decline of patients aged 40–59 years by 36% (from 3357 to 2136 cases; − 80.3 ± 8.4 diagnosis/year; p < 0.001), significant decrease of patients aged 60–79 years by 39% (from 9292 to 5678 cases; − 260.7 ± 18.2 diagnosis/year; p < 0.001) and a significant increase of octogenarians by 24% (from 1983 to 2463 cases; + 25.9 ± 5.3 diagnosis/year; p < 0.001).

### Urethral stricture surgery

**Fig. 3:**
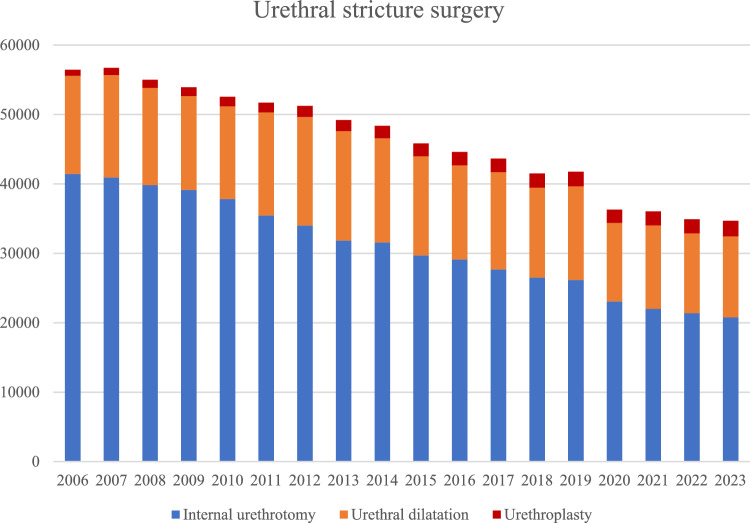
Urethral stricture surgery in German clinics from 2006–2023

A total of 834,476 procedures for USD were analyzed. A significant decline of in-hospital surgical interventions for USD in adult men in Germany was observed from 2006 to 2023 (− 39%; from 56,450 to 34,690 cases; -1403.9 ± 50.3 surgeries/year; p < 0.001). Trends in surgical management of urethral stricture disease over time were visualized in Fig. 3: Internal urethrotomies significantly decreased by 50% (from 41,443 to 20,777 cases; surgeries/year; p < 0.001). Also, urethral dilatations significantly decreased by 17% (from 14,134 to 11,674 cases; − 173.5 ± 46.1 surgeries/year; p = 0.002).

Urethroplasties increased by 156% (from 873 to 2239 cases; + 72.5 ± 5.5 surgeries/year; p < 0.001). This development is depicted in Fig. 2. Table 1 gives detailed information on the three main surgical procedures. The distribution of single-stage and staged urethroplasties remained relatively stable during the study period with 85–90% single-stage and 10–15% staged procedures per year.

**Table 1:** Development of the number of urethral stricture surgeries from 2006 to 2023

Surgical procedure	Case number in 2006	Case number in 2023	Percentage change	Change/year	p value
Internal urethrotomy					
Direct vision (“cold knife”)	23,752	13,838	– 42%	– 641.8 ± 20.6	** < 0.001**
Otis (“cold knife”)	16,873	6664	– 61%	– 617.5 ± 24.1	** < 0.001**
Laser (“hot knife”)	818	275	– 66%	– 43.7 ± 3.4	** < 0.001**
Urethroplasty					
Foreskin	68	326	+ 379%	+ 14.2 ± 1.0	** < 0.001**
Penile skin	93	109	+ 17%	– 0.1 ± 0.6	0.8
Oral mucosa	559	1,234	+ 121%	+ 31.7 ± 5.1	** < 0.001**
Bladder mucosa	9	9	± 0%	– 0.1 ± 0.1	0.9
In vitro oral mucosa	53 (2018)^§^	4	– 92%	– 7.9 ± 3.6	0.09
Undefined	144	557	+ 287%	+ 25.9 ± 0.9	** < 0.001**
Dilatation					
Conventional	14,134	11,575	– 18%	– 175.5 ± 46.3	**0.002**
Drug– coated	17 (2022)*	99	+ 482%	N.a	N.a

A p-value of less than 0.05 was considered significant. Statistically significant differences are highlighted in bold

^*^Year of introduction; ^§^year of peak–introduction in 2013 with 34 cases

Between 2006 and 2023 the decline in internal urethrotomies was more pronounced in younger patients (20-39y: − 48%; 40-59y: − 57%; 60-69y: − 57%) than in older patients (≥ 80y: − 13%). Likewise, urethral dilatations declined in younger patients (20-39y: − 31%; 40-59y: − 33%; 60-69y: − 27%) and increased in octogenarians (+ 46%). Conversely, the increase in urethroplasties was highest among younger patients (20-39y: + 352%; 40-59y: + 94%; 60-79y: + 78%), but also in very old patients a distinct increase despite the small number of cases was observed (≥ 80y: + 229%) (Suppl. Tbl. 3).

## Discussion

The present study investigates contemporary data on USD and associated in-hospital surgical treatments in Germany from 2006 to 2023. Key observations were a significant decrease in USD, a decline in internal urethrotomies and urethral dilatations while the share of urethroplasties significantly increased during the study period.

We observed a decrease by 31% in the diagnosis of USD among adult men. Declining incidence rates in USD were also described by Santucci et al. in 2007 [2]. In contrast, a recent publication showed relatively stable trends in the incidence of USD between 2008 and 2016 in the US [14]. Our results revealed the most significant decrease in postinfectious and posttraumatic strictures with only a modest decline observed in iatrogenic strictures. This small decrease in iatrogenic cases may be attributed to enhanced safety protocols, advances in procedural techniques, and greater adherence to evidence-based practices in urological care, particularly in medical and surgical interventions [15]. The decline in postinfectious (− 84%) and posttraumatic (− 64%) strictures are especially remarkable. These trends likely reflect the widespread use of antibiotics and public health measures aimed at reducing sexually transmitted infections as well as advances in trauma care, including timely surgical repairs and rehabilitation [6, 16]. Furthermore, the reduction in not specified strictures (− 33%) may also indicate improved diagnostic tools and more accurate classification of stricture etiologies. Especially advances in imaging modalities and endoscopic techniques have likely enhanced diagnostic precision, thereby reducing the reliance on non-specific diagnoses.

The age-specific trends in USD showed a non-significant decline for younger patients (20–39 years), likely due to consistent risk factors such as trauma and congenital conditions, while middle-aged (40–59 years) and older (60–79 years) patients demonstrated significant declines, likely reflecting improved preventive and healthcare measures. In contrast, the 24% increase among octogenarians may be attributed to longer life expectancy and possibly a more proactive approach (e.g. TUR-P) in this age group compared to previous decades.

Our analysis showed a decline by 39% for all in-hospital interventions for USD, which aligns with the overall decrease in diagnoses. Internal urethrotomies decreased by 50% and urethral dilatations by 17%, respectively. This trend reflects a move away from less definitive treatment options which are associated with higher recurrence rates. In line with our results, an Australian study between 1994 and 2016 showed a clear shift from repetitive endoscopic procedures towards urethroplasty [17]. Similar, practice patterns among American urologists highlight a shifting paradigm that increasingly favors urethroplasty as the preferred treatment for USD [18]. In another US study, Moynihan et al. reported declining numbers of endoscopic treatments of USD prior to urethroplasties, which could be partly driven by the 2016 publication of guidelines from the American Urological Association (AUA) [19]. We observed a 156% increase in urethroplasties, driven primarily by their low recurrence rates across various techniques and stricture locations, making them a preferred treatment option in recent years. We hypothesize that the more urethroplasties are performed and more surgical experience is gained, the more likely patients are offered open surgery for USD. In that respect, the number of two stage urethroplasties has increased over time, likely also reflecting increasing surgical experience to tackle complex stricture disease.

The worldwide trend towards urethroplasty is likely also a repercussion of changing guidelines on USD over time, as there has been a significant shift towards recommending urethroplasty as the gold standard for the treatment of longer or recurrent strictures. The current EAU guidelines explicitly do not recommend DVIU / dilatation for penile or long (> 2 cm) segment strictures (strength rating: strong) [20]. Generally, urethroplasty should be offered whenever possible as definitive treatment [20].

The age-specific analysis showed a dramatic increase in urethroplasties among young patients (20–39 years, + 352%) indicating a growing trend toward early and definitive management of USD in this population. Notably, there was also a distinct increase among very old patients (≥ 80 years, + 229%). Urethroplasty success may be affected by comorbidities but not age [21]. Therefore, a recent study recommended, that age alone should not be used as an absolute exclusion criterion for men needing urethral reconstruction [21]. The more pronounced decrease in urethrotomies among younger patients (20–39 years, − 48%) compared to older patients (≥ 80 years, − 13%) may indicate a shift in therapeutic goals, prioritizing freedom of recurrence for younger men. The increase in urethral dilatations among very old patients (≥ 80 years, + 46%) likely reflects a preference for less invasive, symptomatic approaches. Specifically, patients who are unfit for complex surgeries may profit from this quick and safe technique.

Interestingly, in our cohort, laser urethrotomy (“hot knife”) showed a greater decline compared to cold knife approaches. Gamal et al. conducted a randomized study comparing “cold knife” and Holmium:YAG laser urethrotomy, finding comparable improvements in maximum urinary flow between the two groups at one-year follow-up [22]. Further, urethroplasty using in vitro oral mucosa declined by 92% with only four cases being performed in Germany in 2023. Osman et al. assessed the progress in producing tissue engineered buccal mucosa as an alternative graft material for urethral stricture repair [23]. The authors described some patients doing well many years post-grafting, while others develop stricture recurrence due to underlying fibrosis formation [23]. Although these options haven’t caught on in daily routine, they might play a more significant role in the future.

In 2022 the drug-coated ballon dilatation of the urethra with Optilume was introduced in Germany. We observed an increase from 17 cases in 2022 to 99 cases in 2023. However, a fair share of patients might have been treated in an outpatient setting, thus not included in this study.

While our study is the first to investigate trends for USD management in Germany over an 18-year time span, certain limitations should be acknowledged. First, the analyzed dataset only includes in-hospital treatments. Therefore, we were not able to cover interventions for USD in an outpatient setting. Hence, a substantial number of urethral dilatations performed as an outpatient procedure might be missed in this analysis. Second, patient characteristics and clinical information on stricture site or length are not part of the dataset. Overall, the lack of patient-level clinical data (e.g. comorbidities, disease severity) limits the scope of the findings. Finally, the quality depends on the accuracy of the documentation by the attending physician. This might explain the overuse of the code N35.9 (not specified) as exact documentation of the etiology is not relevant for remuneration of the case. Generally, reliance on DRG coding may introduce misclassification bias.

## Conclusion

The present study highlights significant changes for USD diagnoses and associated surgical management. We were able to show a declining incidence of USD and the increasing use of urethroplasty reflecting advancements in diagnostic precision and urological surgical techniques. In the future, more population-based data on USD management, ideally combined with patients-reported outcomes, would enhance our knowledge on current trends and help us assess the quality of care for this complex disease.

## Supplementary Information

Below is the link to the electronic supplementary material.

Supplementary file1 (DOCX 16 KB)

## Data Availability

The data used for this analysis is publicly available (hospital quality reports).
