# Supplementary material for: Increasing utilization of urethroplasty for male urethral stricture disease: analysis of in-hospital interventions in Germany from 2006 to 2023
Source: Int Urol Nephrol. 2025 Apr 28;57(11):3559–65. doi: 10.1007/s11255-025-04487-1 (PMC12504392; doi:10.1007/s11255-025-04487-1)
Supplement: Supplementary file 1 — Supplementary file1 (DOCX 16 KB) [file 11255_2025_4487_MOESM1_ESM.docx]

**Supplements**

**Supplementary Table 1.** Urethral stricture disease ICD-10 codes.

| **Description** | **ICD-10 code** |
| --- | --- |
| Posttraumatic urethral stricture | N35.0 |
| Postinfectious urethral stricture | N35.1 |
| Urethral stricture, not specified | N35.8, N35.9 |
| Urethral stricture after medical intervention (iatrogenic) | N99.1 |

**Supplementary Table 2:** Description of urethral stricture disease-related OPS codes.

| **Description** | **OPS code** |
| --- | --- |
| **Internal urethrotomy** | |
| Internal urethrotomy (Otis) | 5-585.0 |
| Direct vision internal urethrotomy with cold knife (DVIU, Sachse) | 5-585.1 |
| Direct vision internal urethrotomy with laser (DVIU, Sachse) | 5-585.2 |
| **Urethral dilatation** | |
| *Dilatation of the urethra* | 8-139.0 |
| Dilatation of the urethra – without fluoroscopic guidance | 8-139.01 |
| Dilatation of the urethra – under fluoroscopic guidance | 8-139.02 |
| *Ballondilatation of the urethra* | 8-139.1 |
| Ballondilatation of the urethra – without drug-coated ballon | 8-139.10 |
| Ballondilatation of the urethra – with drug-coated ballon | 8-139.11 |
| Ballondilatation of the urethra, not specified | 8-139.x |
| Ballondilatation of the urethra, not specified | 8-139.y |
| **Urethroplasty** | |
| *Urethroplasty – single stage procedures* | 5-584.7 |
| With prepucial skin graft | 5-584.70 |
| With penile skin graft | 5-584.71 |
| With buccal mucosa graft | 5-584.72 |
| With urinary bladder mucosa graft | 5-584.73 |
| With in vitro buccal mucosa graft | 5-584.74 |
| Not specified | 5-584.7x |
| *Urethroplasty – two / multiple stage procedures* | 5-584.8 |
| With prepucial skin graft | 5-584.80 |
| With penile skin graft | 5-584.81 |
| With buccal mucosa graft | 5-584.82 |
| With urinary bladder mucosa graft | 5-584.83 |
| With in vitro buccal mucosa graft | 5-584.84 |
| Not specified | 5-584.8x |

**Supplementary Table 3.** Development of the age distribution regarding urethral stricture surgeries from 2006 to 2023

| **Age groups** | | **case number in 2006** | **case number in 2023** | **percentage change** | **change/year** | **p value** |
| --- | --- | --- | --- | --- | --- | --- |
| Internal urethrotomy | 20 – 39 years | 1,563 | 809 | -48% | -38.9±2.7 | **<0.001** |
|  | 40 – 59 years | 6,151 | 2,630 | -57% | -219.1±5.9 | **<0.001** |
|  | 60 – 79 years | 27,348 | 11,769 | -57% | -983.0±24.1 | **<0.001** |
|  | 80+ years | 6,381 | 5,569 | -13% | -62.0±9.3 | **<0.001** |
| Urethroplasty | 20 – 39 years | 221 | 1,000 | +352% | +48.6±1.9 | **<0.001** |
|  | 40 – 59 years | 295 | 573 | +94% | +13.8±2.7 | **<0.001** |
|  | 60 – 79 years | 336 | 597 | +78% | +7.6±2.9 | **0.017** |
|  | 80+ years | 21 | 69 | +229% | +2.5±0.3 | **<0.001** |
| Dilatation | 20 – 39 years | 776 | 532 | -31% | -11.0±3.1 | **0.003** |
|  | 40 – 59 years | 2,696 | 1,806 | -33% | -59.9±104.1 | **<0.001** |
|  | 60 – 79 years | 8,551 | 6,259 | -27% | -165.4±26.8 | **<0.001** |
|  | 80+ years | 2,111 | 3,077 | +46% | +62.8±9.8 | **<0.001** |
